# Structural and biochemical analyses of a *Clostridium perfringens* sortase D transpeptidase

**DOI:** 10.1107/S1399004715009219

**Published:** 2015-06-30

**Authors:** Randy Suryadinata, Shane A. Seabrook, Timothy E. Adams, Stewart D. Nuttall, Thomas S. Peat

**Affiliations:** a Manufacturing Flagship, Commonwealth Scientific and Industrial Research Organisation, 343 Royal Parade, Parkville, Victoria 3052, Australia

**Keywords:** sortase D, spore-forming Gram-positive bacterium, LPQTGS sorting motif

## Abstract

The assembly and anchorage of various pathogenic proteins on the surface of Gram-positive bacteria is mediated by the sortase family of enzymes. These cysteine transpeptidases catalyze a unique sorting signal motif located at the C-terminus of their target substrate and promote the covalent attachment of these proteins onto an amino nucleophile located on another protein or on the bacterial cell wall. Each of the six distinct classes of sortases displays a unique biological role, with sequential activation of multiple sortases often observed in many Gram-positive bacteria to decorate their peptidoglycans. Less is known about the members of the class D family of sortases (SrtD), but they have a suggested role in spore formation in an oxygen-limiting environment. Here, the crystal structure of the SrtD enzyme from *Clostridium perfringens* was determined at 1.99 Å resolution. Comparative analysis of the *C. perfringens* SrtD structure reveals the typical eight-stranded β-barrel fold observed in all other known sortases, along with the conserved catalytic triad consisting of cysteine, histidine and arginine residues. Biochemical approaches further reveal the specifics of the SrtD catalytic activity *in vitro*, with a significant preference for the LPQTGS sorting motif. Additionally, the catalytic activity of SrtD is most efficient at 316 K and can be further improved in the presence of magnesium cations. Since *C. perfringens* spores are heat-resistant and lead to foodborne illnesses, characterization of the spore-promoting sortase SrtD may lead to the development of new antimicrobial agents.

## Abstract 2

The structure of *C. perfringens* sortase D was determined at 1.99 Å resolution. Comparative biochemical and structural analyses revealed that this transpeptidase may represent a new subclass of the sortase D family.

## 1. Introduction   

*Clostridium perfringens* is an anaerobic, spore-forming bacterium found in a wide range of environmental conditions including soil, marine sediments and the intestinal tract of humans and other vertebrates (McClane, 2007 ▸; Brynestad & Granum, 2002 ▸; Grass *et al.*, 2013 ▸). This highly pathogenic Gram-positive bacterium is the second most common cause of foodborne diseases in the US, with an estimated one million reports each year (Scallan *et al.*, 2011 ▸). Additionally, *C. perfringens* isolates can be responsible for the development of non-foodborne human gastrointestinal diseases, including sporadic diarrhoea and antibiotic-associated diarrhoea (Collie *et al.*, 1998 ▸). The pathogenesis of *C. perfringens*-derived foodborne illnesses typically originates from germination of the spores in raw and cooked food under oxygen-limiting conditions (Jenuja *et al.*, 2010 ▸). Significantly, the formation of spores correlates with the survival mode of the organism, which allows it to resist extreme temperatures, including heat treatment and refrigeration (Jenuja *et al.*, 2010 ▸; Strong *et al.*, 1966 ▸; Traci & Duncan, 1974 ▸). Once digested, *C. perfringens* isolates germinate in the intestinal tract, where they produce *C. perfringens* enterotoxin (CPE), resulting in gastrointestinal illnesses (Jenuja *et al.*, 2010 ▸).

The exact mechanisms contributing to the pathogeneicity of *C. perfringens* isolates remain unclear, with a number of factors identified to promote the survival of the organism (Orsburn *et al.*, 2008 ▸). In common with many pathogenic Gram-positive bacteria, *C. perfringens* displays and anchors a diverse array of surface proteins on its cell wall with functions such as adaptation to extreme environmental conditions, evasion of the host immune system, and virulence (Hendrickx *et al.*, 2011 ▸; Marraffini & Schneewind, 2006 ▸). The covalent attachment of many of these proteins is mediated by the so-called sortase enzymes, a unique family of membrane-bound, cysteine transpeptidases which were first identified in *Staphylococcus aureus* (Mazmanian *et al.*, 1999 ▸). Mechanistically, the sortase catalytic activity is best illustrated for *S. aureus* sortase A, where the enzyme recognizes a unique pentapeptide sorting motif (Leu-Pro-*X*-Thr-Gly, LP*X*TG, where *X* denotes any amino acid) within the cell-wall sorting signal (CWSS) region located at the carboxyl-terminus (Kruger *et al.*, 2004 ▸; Ton-That *et al.*, 1999 ▸). Upon recognition, sortase A (SrtA) cleaves the Thr-Gly peptide bond, leading to loss of the C-terminal glycine and the formation of a thioacyl intermediate (Kruger *et al.*, 2004 ▸; Ton-That *et al.*, 1999 ▸). Subsequently, the presence of oligo-glycine acts as a nucleophile to dissociate the acyl-enzyme intermediate and promote sortase-assisted covalent coupling of the free amino group of the oligo-glycine to the Thr carboxyl group (Suree *et al.*, 2009 ▸; Weiner *et al.*, 2010 ▸).

Beyond the versatile housekeeping enzyme SrtA, the sortase enzymes can phylogenetically be characterized into five other distinct classes (Suree *et al.*, 2007 ▸). The latter classes display more specialized roles. For example, members of the sortase B family are catalytically important for iron acquisition (Maresso *et al.*, 2006 ▸; Maresso & Schneewind, 2006 ▸; Mazmanian *et al.*, 2002 ▸, 2003 ▸) and class C sortases are predominantly responsible for the assembly of pili, which are involved in microbial adhesion and biofilm formation (Spirig *et al.*, 2011 ▸; Cozzi *et al.*, 2012 ▸, 2013 ▸; Khare *et al.*, 2011 ▸; Manzano *et al.*, 2008 ▸; Neiers *et al.*, 2009 ▸; Persson, 2011 ▸; Wu *et al.*, 2012 ▸). Much less is known about the class D, E and F enzymes. Class D sortases are thought to induce spore formation in an oxygen-limiting environment (Marraffini & Schneewind, 2006 ▸, 2007 ▸), and the study of *Bacillus anthracis* SrtD revealed an exclusive preference towards the Leu-Pro-Asn-Thr-Ala (LPNTA) signal motif (Marraffini & Schneewind, 2006 ▸). Interestingly, *B. anthracis* also expresses the class A sortases which recognize the canonical LP(A/N/K)TG signal motif. Despite the signal motifs only differing slightly in their sequences, both *B. anthracis* SrtA and SrtD function non­redundantly, indicating evolved specificity towards the respective signal motifs (Marraffini & Schneewind, 2006 ▸). Functional analyses further revealed that *B. anthracis* SrtD functions at different stages of sporulation, including the attachment of the acidic surface protein BasH to the peptidoglycans of developing forespores (precursor spores; Marraffini & Schneewind, 2006 ▸) and the presentation of the BasI surface protein on the envelope of pre-divisional sporulating cells (Marraffini & Schneewind, 2007 ▸).

In the current study, we present the crystal structure of a *C. perfringens* transpeptidase which belongs to the class D family of sortases, suggesting a potential role of this enzyme in *C. perfringens* spore formation. Biochemically, the recombinant *C. perfringens* SrtD (*Cp*SrtD) is catalytically active, with a high preference for the Leu-Pro-Gln-Thr-Gly-Ser (LPQTGS) signal motif. Additionally, *Cp*SrtD catalytic activity is also dependent on a metal cation, with the presence of magnesium appearing to enhance *Cp*SrtD catalysis towards the LPQTGS signal motif. The structure of *Cp*SrtD is distinct from the previously reported NMR structure of *B. anthracis* sortase D (Robson *et al.*, 2012 ▸), leading us to propose *C. perfringens* sortase D as a new subclass of the D-type sortase family.

## 2. Materials and methods   

### 2.1. Cloning, expression and purification of *Cp*SrtD   

Codon-optimized *C. perfringens* sortase D (*Cp*SrtD; CPE_RS01475) cDNA encoding residues 23–187 was synthesized at GeneArt AG and cloned into the pET-28a expression vector (Novagen) at the NdeI and XhoI restriction sites to generate an N-terminally His_6_-tagged recombinant protein.

*E. coli* Rosetta BL21 (DE3) cells transformed with the pET-28a-*Cp*SrtD expression plasmid were incubated in 2YT medium with 50 µg ml^−1^ kanamycin at 310 K until the OD_600_ reached 0.6–0.8. Recombinant protein expression was induced by adding 1 m*M* IPTG for 3 h at 310 K. The cells were collected by centrifugation and resuspended in lysis buffer [50 m*M* HEPES pH 7.5, 300 m*M* NaCl, 5%(*v*/*v*) glycerol, 15 m*M* imidazole, 10 m*M* β-mercaptoethanol, 2 m*M* MgCl_2_, 0.0025 units µl^−1^ Benzonase (Novagen), 250 µg ml^−1^ lysozyme and protease-inhibitor cocktail (Roche)]. Lysates were passed three times through an EmulsiFlex-C5 cell crusher (Avestin) at 103.4 MPa and 277 K, centrifuged and the clarified lysates were run over a 5 ml His-Trap FF IMAC chromatography column (GE). Following extensive washes with buffer *A* (50 m*M* HEPES pH 7.5, 300 m*M* NaCl, 15 m*M* imidazole) to remove unbound proteins, recombinant His_6_-*Cp*SrtD was collected using buffer *B* (50 m*M* HEPES pH 7.5, 300 m*M* NaCl, 250 m*M* imidazole). The recombinant protein was further purified on a Superdex 75 26/60 size-exclusion chromatography column (GE; Supplementary Fig. S1); approximately 60 mg l^−1^ recombinant enzyme in 50 m*M* HEPES pH 7.5, 150 m*M* NaCl, 5%(*v*/*v*) glycerol was recovered and was stored as aliquots at 193 K until used for downstream analyses.

### 2.2. Differential scanning fluorimetry (DSF)   

Protein stability was determined across a series of conditions encompassing a range of different buffers/pH values and salts as previously described (Seabrook & Newman, 2013 ▸). Recombinant *Cp*SrtD was found to be most stable in a buffer consisting of 50 m*M* MES pH 6.5, 200 m*M* NaCl (Supplementary Fig. S2).

### 2.3. Crystallization   

Crystallization experiments of recombinant *Cp*SrtD were set up at both 281 and 293 K using the Netherlands Cancer Institute (NKI) dual screen set (Newman *et al.*, 2005 ▸). Recombinant *Cp*SrtD was prepared at a concentration of 20 mg ml^−1^ in a 50 m*M* MES pH 6.5, 200 m*M* NaCl buffer formulation, and sitting-drop vapour-diffusion experiments were then set up using 200 nl protein solution and 200 nl reservoir solution. Protein crystals successfully grew under several conditions at both 281 and 293 K, with the best buffer formulation for growing native *Cp*SrtD crystals consisting of 200 m*M* ammonium acetate, 100 m*M* bis-tris chloride pH 5.5, 25%(*w*/*v*) PEG 3350, yielding crystals after 1 d of incubation at 281 K. Under this condition, the protein crystal adopts a thin plate morphology with dimensions of approximately 350 × 600 µm (Supplementary Fig. S3*a*).

### 2.4. Data collection and structural determination   

360 1° images were obtained on the MX-2 microfocus beamline at the Australian Synchrotron from a crystal that had been cryocooled to 100 K. The reflections were indexed using *XDS* (Kabsch, 2010 ▸) and scaled using *AIMLESS* (Evans, 2011 ▸). *ClustalW* was used to align the *C. perfringens* sortase sequence with the sequence from PDB entry 3g66 (Neiers *et al.*, 2009 ▸), and *CHAINSAW* (Stein, 2008 ▸) was then used with PDB entry 3g66 (with an estimated sequence identity of 21%) to obtain a model for *Phaser* (McCoy *et al.*, 2007 ▸), which was used to obtain the initial phases. The initial *Phaser* output LLG was 21.5, with a *Z*-score of 5.8. Two molecules were placed in the asymmetric unit and the final output values for the solution were LLG = 90.7, TFZ = 7.2, with an *R* value of 56.8. The model was initially rebuilt using *Buccaneer* (Cowtan, 2006 ▸) and subsequently rebuilt manually using *Coot* (Emsley *et al.*, 2010 ▸) and refined using *REFMAC* (Murshudov *et al.*, 2011 ▸). The data were 99.5% complete to a resolution of 1.99 Å and the final model had an *R*_work_ of 17.6% and an *R*_free_ of 21.3% (see Table 1 ▸ for crystallographic statistics). According to the PDB report, 97% of the residues are in the most favoured region of the Ramachandran plot and 3% are in the allowed region, with no outliers.

### 2.5. *In vitro* thioacyl intermediate formation   

A solution containing 70 µ*M* *Cp*SrtD was incubated with a 15 µ*M* solution of a peptide comprising of the first 16 amino-acid residues of amyloid-β (A*β*_1–16_) fused at the C-terminus to a variety of sortase signal motifs in the presence of MES reaction buffer (50 m*M* MES pH 6.5, 200 m*M* NaCl, 1 m*M* TCEP) for 3 h at 316 K. The reaction was quenched by adding nonreducing NuPAGE loading buffer (Life Technologies). Following SDS–PAGE, resolved protein samples were transferred onto nitrocellulose membranes for Western blot analyses of thioacyl intermediate formation using an antibody against A*β* (WO2). Equal loading was determined by Western blot using anti-His_5_ antibody (Qiagen).

To analyze the impact of different metal ions on the catalytic activity of *Cp*SrtD, recombinant protein was first incubated with 100 m*M* EDTA for 2 h at room temperature (RT). EDTA-treated *Cp*SrtD was then diluted tenfold before being tested for catalytic activity in the presence of 10 m*M* metal ions for 3 h at 316 K. The reaction was quenched by the addition of nonreducing NuPAGE loading buffer. SDS–PAGE and subsequent Western blot analyses were then performed as above.

### 2.6. Dynamic light scattering (DLS)   

A 20 µl aliquot containing 20 mg ml^−1^ *Cp*SrtD was dispensed into each well of a black 384-well microplate with an optically clear base (Corning). Measurements were collected at 293 K using 5 s acquisitions and allowing the attenuation and laser power to be automatically set by the DLS system (DynaPro Plate Reader, Wyatt). The resulting distributions were derived from regularization fits to the average of 50 correlation curves using the *DYNAMICS* software (Wyatt) and are displayed as the intensity of light scattered as a function of the hydrodynamic radius (Supplementary Fig. S4).

## 3. Results   

### 3.1. Overall structure of *C. perfringens* sortase   

**Figure 1:**
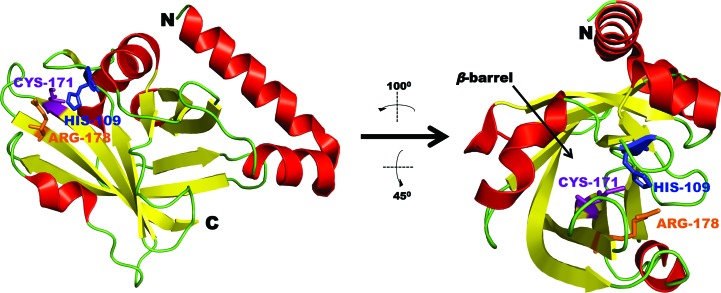
The overall structure of *C. perfringens* sortase D. The secondary structure of *C. perfringens* sortase D monomer *A* is represented by red 3_10_-helices and α-­helices and yellow β-strands (PDB entry 4d70). The N- and C-termini of the enzyme are indicated. The conserved catalytic triad consisting of His109 (blue), Cys171 (purple) and Arg178 (orange) is shown. The yellow β-strands form the β-barrel structure which is typically observed in the sortase family of enzymes (right-hand side). Figures were generated using *PyMOL* (v.1.5.0.4; Schrödinger).

**Figure 2:**
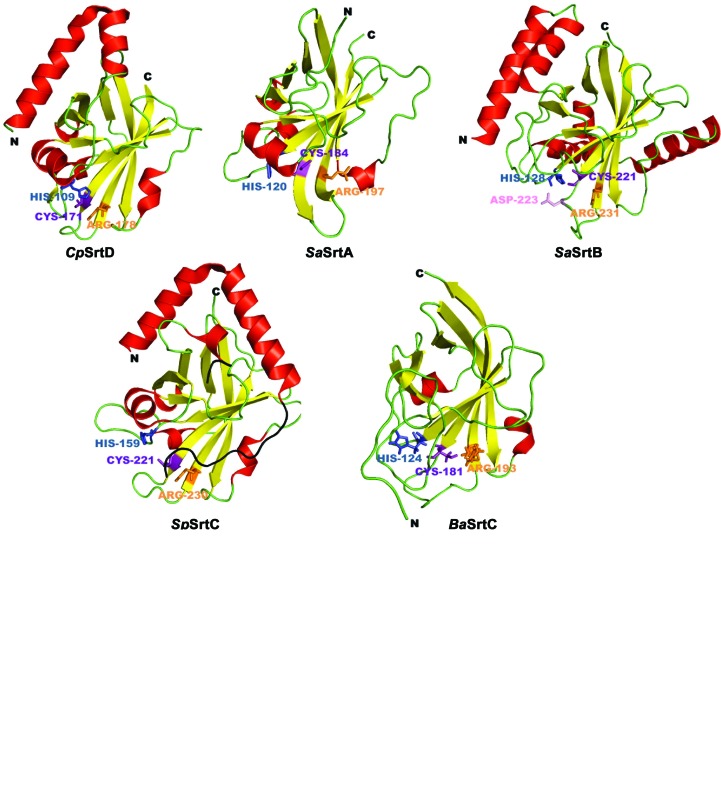
Comparison between *C. perfringens* sortase D and representatives of classes A to D of transpeptidases. (*a*) The secondary structures of *C. perfringens* sortase D (*Cp*SrtD; PDB entry 4d70), along with *S. aureus* sortase A (*Sa*SrtA; PDB entry 1t2p; Zong *et al.*, 2004 ▸), *S. aureus* sortase B (*Sa*SrtB; PDB entry 1ng5; Zhang *et al.*, 2004 ▸), *S. pneumoniae* sortase C-2 (*Sp*SrtC2; PDB entry 3g66; Neiers *et al.*, 2009 ▸) and *B. anthracis* sortase D (BaSrtC; PDB entry 2ln7; Robson *et al.*, 2012 ▸) are represented by red 3_10_-helices and α-helices and yellow β-strands. The ‘lid’ structure of the class C sortases is depicted in black, while the conserved cysteine, histidine and arginine residues are shown in purple, blue and orange, respectively. The conserved aspartate residue in *Sa*SrtB is shown in pink. Figures were generated using *PyMOL*. (*b*) Sequence alignment between full-length *Cp*SrtD, *Sa*SrtA, *Sa*SrtB, *Sp*SrtC2 and *Ba*SrtC, revealing identical amino acids (highlighted in red) and residues with high degree of similarity (highlighted in yellow). The conserved cysteine, histidine and arginine are indicated by asterisks. Sequence alignment was generated using *ClustalW Omega* (Goujon *et al.*, 2010 ▸; Sievers *et al.*, 2011 ▸).

In this study, we report the crystal structure of a *C. perfringens* sortase that was solved at 1.99 Å resolution by molecular replacement (Table 1 ▸). Recombinant *C. perfringens* sortase crystallized in space group *P*2_1_ with two molecules in the crystallographic asymmetric unit (Supplementary Fig. S3*b*). The final maps derived from the X-ray data showed clear density for 160 residues (28–187), only lacking the first five residues along with the N-terminal hexahistidine tag and thrombin cleavage site. Alignment of the two monomers revealed a r.m.s.d. value of less than 0.53 Å, with a slight conformational difference of the turn within the α1–α2 helix–turn–helix structure (Supplementary Fig. S3*c*). Each monomer displayed the typical eight β-strands that form a β-barrel structure (Fig. 1 ▸). This distinct barrel structure is also present in other sortases (Fig. 2 ▸ *a*) and serves as a hallmark of this family of enzymes. A second distinctive feature observed in all sortase enzymes is the surface presentation of the conserved active site, comprising of a catalytic cysteine that is surrounded by a histidine and an arginine residue (Fig. 2 ▸ *a*). Previous studies have demonstrated that the presence of both histidine and arginine are necessary for efficient catalysis by the conserved cysteine residue (Frankel *et al.*, 2007 ▸; Clancy *et al.*, 2010 ▸). These key residues are also present on the surface of *C. perfringens* sortase, with the catalytic cysteine located within the β7 strand at position 171 (Fig. 1 ▸). The adjacent arginine is found in the β8 strand at position 178, while the histidine is positioned within the β3–α4 loop at residue 109 (Fig. 1 ▸).

Initial sequence analyses revealed that the *C. perfringens* sortase belongs to the class D subfamily 5 of transpeptidases (Dramsi *et al.*, 2005 ▸), which is also referred to as class E of sortases (SrtE; Spirig *et al.*, 2011 ▸). Comparative sequence analyses of the *C. perfringens* sortase D with the previously identified *S. aureus* sortase A (*Sa*SrtA) and sortase B (*Sa*SrtB), *Streptococcus pneumoniae* sortase C-2 (*Sp*SrtC2) and *B. anthracis* sortase D (*Ba*SrtC) revealed approximately 18, 25, 26 and 28% identity in their amino-acid sequences (Fig. 2 ▸ *b*). The sparse similarity between the amino-acid sequences of the *C. perfringens* sortase and members of classes A–D of the sortase family further highlights that the *C. perfringens* transpeptidase may belong to a new class.

### 3.2. *C. perfringens* sortase recognizes the LPQTGS signal motif for transpeptidation   

**Figure 3:**
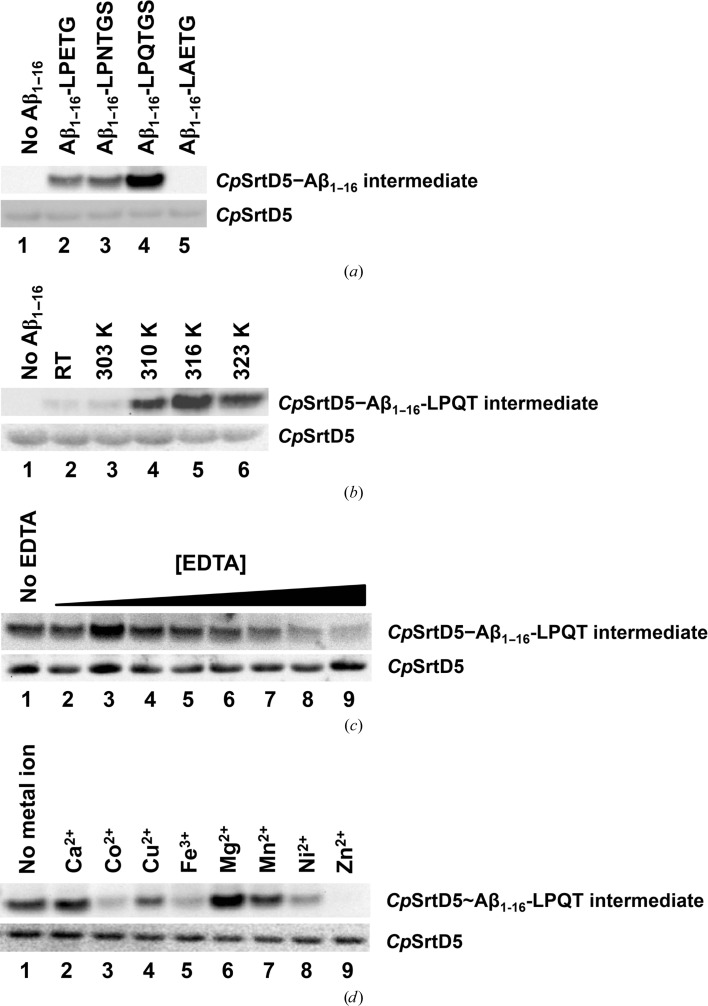
*Cp*SrtD recognizes and cleaves the LPQTGS sorting motif *in vitro*. (*a*) Recombinant *Cp*SrtD was incubated in the absence (lane 1) or presence of Aβ_1–16_ peptides fused to LPETG (lane 2), LPNTGS (lane 3), LPQTGS (lane 4) or LAETG (lane 5) sorting motifs. (*b*) Catalytic efficiency of recombinant *Cp*SrtD towards Aβ_1–16_-LPQTGS substrate was measured at room temperature (RT, lane 2), 303 K (lane 3), 310 K (lane 4), 316 K (lane 5) or 323 K (lane 6). (*c*) *Cp*SrtD was pre-incubated in the absence (lane 1) or presence of EDTA at a concentration of 1 m*M* (lane 2), 2 m*M* (lane 3), 5 m*M* (lane 4), 10 m*M* (lane 5), 20 m*M* (lane 6), 50 m*M* (lane 7), 100 m*M* (lane 8) or 200 m*M* (lane 9). EDTA-treated *Cp*SrtD was then incubated with Aβ_1–16_-LPQTGS and the ability to form the thioacyl intermediate was measured. (*d*) EDTA-treated (100 m*M*) *Cp*SrtD was incubated with Aβ_1–16_-LPQTGS in the absence of metal ions (lane 1) or the presence of 10 m*M* Ca^2+^ (lane 2), Cu^2+^ (lane 3), Co^2+^ (lane 4), Fe^3+^ (lane 5), Mg^2+^ (lane 6), Mn^2+^ (lane 7), Ni^2+^ (lane 8) or Zn^2+^ (lane 9). The formation of thioacyl intermediate in (*a*), (*b*) and (*c*) was analysed by Western blot using mouse α-­Aβ (WO2) antibody (top panels), and equal loading was assessed using mouse α-His_5_ antibody (bottom panels).

One notable feature that is observed in most sortases is their ability to preferentially recognize a specific signal motif for catalysis. To identify the signal motif preferred by *C. perfringens* sortase to achieve efficient catalysis, we performed a series of *in vitro* transpeptidation reactions using a substrate that consists of the first 16 amino-acid residues of the amyloid-β (Aβ_1–16_) peptide fused at the C-terminus with the LPETG, LPNTGS, LPQTGS or LAETG sorting motifs. This panel of substrates represents signal motifs that are recognized by the different classes of sortase family, including class A (LPETG), class D (LPNTGS and LPQTGS) and class E (LAETG). Western blot analyses using anti-Aβ antibody to detect the *Cp*SrtD–substrate thioacyl intermediate revealed no *Cp*SrtD catalytic activity towards the class E signal motif (Fig. 3 ▸ *a*, lane 5, top panel) and minimal activity towards either the LPETG (Fig. 3 ▸ *a*, lane 2, top panel) or LPNTGS (Fig. 3 ▸ *a*, lane 3, top panel) signal motifs. In contrast, recombinant *Cp*SrtD showed a strong preference towards the LPQTGS motif (Fig. 3 ▸ *a*, lane 4, top panel).

### 3.3. *Cp*SrtD catalysis is temperature-dependent   

Heat-resistant *C. perfringens* spores can be produced at a faster, more efficient rate by incubating the isolates at 316 K (Garcia-Alvarado *et al.*, 1992 ▸). To determine the optimal temperature at which *C. perfringens* SrtD achieves maximal transpeptidation activity *in vitro*, we analyzed the efficiency of the enzyme to catalyze the formation of the thioacyl intermediate at different temperatures (Fig. 3 ▸ *b*). Recombinant *Cp*SrtD is highly inefficient in forming a thioacyl intermediate with the Aβ_1–16_-LPQTGS substrate when incubated at RT (lane 2) or at 303 K (lane 3). However, the transpeptidase activity of the enzyme can be improved by incubating the *Cp*SrtD at higher temperatures (Fig. 3 ▸ *b*, lanes 4–6), with the maximal catalytic efficiency being observed at 316 K (lane 5).

### 3.4. *Cp*SrtD activity is dependent on the presence of metal cation   

Previous studies have demonstrated that *S. aureus* SrtA requires Ca^2+^ ion for its catalytic activity (Naik *et al.*, 2006 ▸). To assess whether *Cp*SrtD activity is also dependent on a metal cation, we first investigated the effect of EDTA on the basal activity of the enzyme. Addition of EDTA reduced the ability of *Cp*SrtD to form thioacyl intermediates with the Aβ_1–16_-LPQTGS substrate in a concentration-dependent manner (Fig. 3 ▸ *c*), indicating metal-ion-dependent catalysis. Interestingly, only EDTA at concentrations of 20 m*M* and higher demonstrated sufficient chelating properties which lead to a reduced *Cp*SrtD transpeptidase activity (Fig. 3 ▸ *c*, lanes 6–9), suggesting the possibility of a tightly bound metal cation to the enzyme.

To further identify the specific metal cation(s) responsible for catalysis, we selected and examined the effect of a panel of divalent and trivalent cations on *Cp*SrtD-mediated transpeptidation (Fig. 3 ▸ *d*). The addition of 10 m*M* CaCl_2_ (lane 2) or MnCl_2_ (lane 7) had no impact on the basal activity of *Cp*SrtD, while the presence of CoCl_2_ (lane 3), CuCl_2_ (lane 4), FeCl_3_ (lane 5) or NiCl_2_ (lane 8) reduced the ability of *Cp*SrtD to form thioacyl intermediates. Interestingly, 10 m*M* ZnCl_2_ (lane 9) completely inhibits *Cp*SrtD transpeptidase activity. In contrast, the addition of MgCl_2_ increases *Cp*SrtD catalytic activity as demonstrated by the increased amount of thioacyl intermediate (lane 6), suggesting the potential importance of the Mg^2+^ cation for *Cp*SrtD activation and catalysis.

## 4. Discussion   

A previous report suggested that the *C. perfringens* sortase described in this study belongs to the class E family of sortases (Spirig *et al.*, 2011 ▸). At the structural level, recombinant *C. perfringens* sortase demonstrated the classic β-barrel configuration found in all sortase enzymes and displays the conserved catalytic cysteine at position 171 flanked by histidine and arginine residues (Fig. 1 ▸). Despite the previous report, our biochemical data indicate that the *C. perfringens* sortase does not belong to the class E family of transpeptidases owing to its inability to recognize and catalyze the LAETG motif (Fig. 3 ▸ *a*, lane 5), the sorting signal motif preferred by this class of enzymes (Duong *et al.*, 2012 ▸). Instead, the recombinant *C. perfringens* sortase demonstrated efficient catalysis towards a class D signal motif, LPQTGS (Fig. 3 ▸ *a*, lane 4), highlighting the possibility that this *C. perfringens* sortase belongs to the class D family of enzymes (*Cp*SrtD) that are responsible for spore formation under anaerobic conditions. This notion was further supported by additional biochemical analyses demonstrating that *Cp*SrtD is most efficient in catalysis at 316 K (Fig. 3 ▸ *b*), which is the temperature reported for optimally inducing spore formation in *C. perfringens* isolates (Garcia-Alvarado *et al.*, 1992 ▸). Our findings on *Cp*SrtD substrate selectivity are also supported by analysis of the *C. perfringens* strain 13 genome, which revealed that the sortase gene is clustered in the same operon as a hypothetical cell-wall anchor protein (CPE_RS01465) which possesses a C-terminal LPQTGS signal motif, thus underlining the possibility of the LPQTGS motif as one of the natural substrates of this enzyme (Shimizu *et al.*, 2002 ▸).

**Figure 4:**
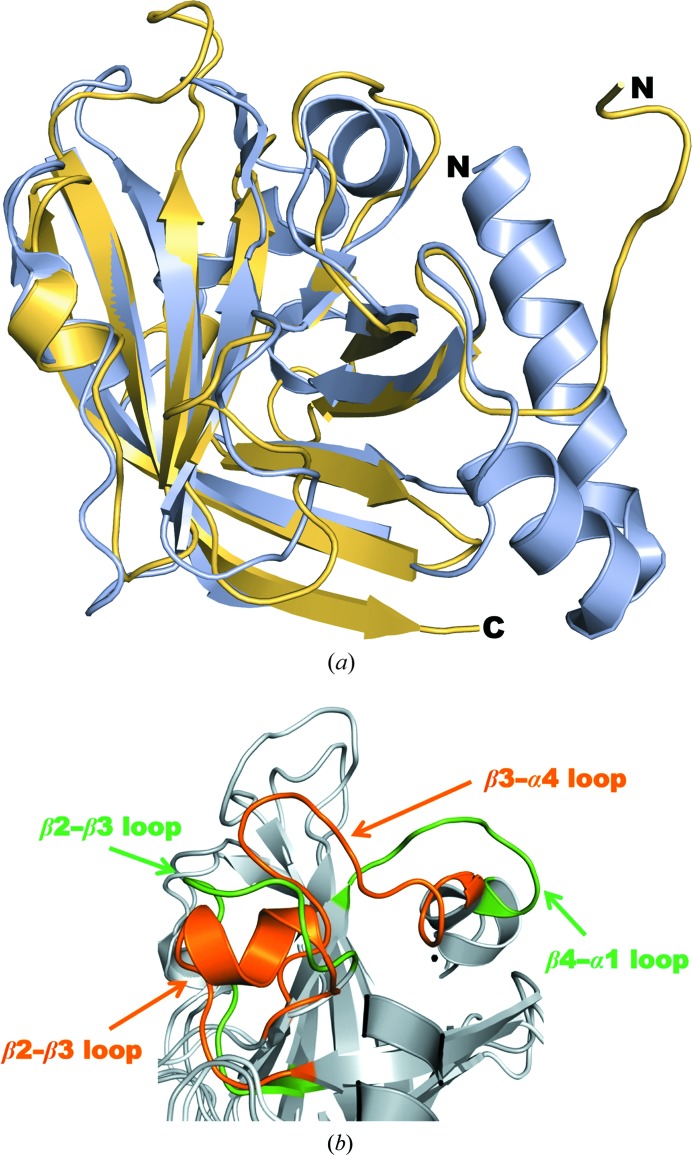
*C. perfringens* sortase D is distinct from the class D enzyme isolated from *B. anthracis*. (*a*) Superposition of the *C. perfringens* (blue; PDB entry 4d70) and *B. anthracis* (gold; PDB entry 2ln7) sortase D structures. (*b*) Comparison of the distinctive loops revealing the presence of a two-turn *α*-helix within the *C. perfringens* sortase D (orange) β2–β3 loop that is not present in the *B. anthracis* SrtD loop (green). The β2–β3 and β4–H1 loops are found to be disordered in the PDB structure 2ln7 but well ordered in the *C. perfringens* sortase D structure. Figures were generated using *PyMOL*.

Comparative sequence analyses revealed that *C. perfringens* sortase D is relatively distinct from the previously reported class D sortase isolated from *B. anthracis* (unfortunately referred to as *Ba*SrtC; Robson *et al.*, 2012 ▸), with a limited 28% identity in their amino-acid sequence (Fig. 2 ▸ *b*). Superposition of the secondary structures reveals further differences between the two enzymes, with a calculated r.m.s.d. of 1.7 Å (Fig. 4 ▸ *a*). The most notable difference is the presence of N-terminal α-helices in *C. perfringens* SrtD which are absent in the *B. anthracis* SrtD structure. Previously, only class B and C sortases have been observed to display long N-terminal α-helices (Fig. 2 ▸ *a*). The N-terminal region of class C sortases plays an important role in catalysis, in which the α-helices flank a flexible loop region that form the so-called ‘lid’ structure (Fig. 2 ▸ *a*), which is thought to be responsible in controlling the access of substrates to the SrtC catalytic site (Khare *et al.*, 2011 ▸; Manzano *et al.*, 2009 ▸; Neiers *et al.*, 2009 ▸). Within the ‘lid’ structure in all class C sortases lies the conserved ‘lid’ domain consisting of Asp-Pro-Try/Trp/Phe (DPY/W/F; Cozzi *et al.*, 2013 ▸; Khare *et al.*, 2011 ▸; Manzano *et al.*, 2009 ▸), and a point mutation of the key residue within the ‘lid’ domain is necessary for activation of the class C sortases *in vitro* (Cozzi *et al.*, 2013 ▸). In contrast, recombinant *Cp*SrtD is catalytically active in the wild-type form *in vitro* (Fig. 3 ▸), and sequence analysis revealed that the conserved ‘lid’ domain is not present in this enzyme (Fig. 2 ▸ *b*). Therefore, it is possible that the N-terminal α-helices present in *C. perfringens* sortase D possess different, albeit unknown, function(s). The role of the N-terminal α-helices in sortase B is also unknown; however, both *S. aureus* and *B. anthracis* sortase B can be distinguished from the other classes of sortases, including *C. perfringens* sortase D, by the presence of an additional conserved residue within the active site (Jacobitz *et al.*, 2014 ▸; Zhang *et al.*, 2004 ▸). These class B sortases possess a conserved aspartate (at position 223 in *Sa*SrtB; Fig. 2 ▸ *a*) which has a suggested role in controlling substrate specificity without affecting the overall transpeptidation activity (Jacobitz *et al.*, 2014 ▸). The overall structure of sortase B represents a nearly equal distribution of α and β structures, with eight β-strands forming the β-barrel core structure surrounded by several long and short α-helices (Fig. 2 ▸ *a*). Conversely, the *S. aureus* sortase A structure is predominantly made up of loops that connect the β-barrel structure (Fig. 2 ▸ *a*). Therefore, unlike the N-terminal portion of *Cp*SrtD and sortases B and C, where it is largely composed of a helix–turn–helix, the N-terminus of *Sa*SrtA is unstructured (Fig. 2 ▸ *a*). It is interesting to note that in contrast to the other classes of sortases, the N-terminus of *Sa*SrtA is positioned on the opposite side to the active site (Fig. 2 ▸ *a*). This indicates the natural orientation of *Sa*SrtA on the bacterial cell wall, where it is likely that the active site is exposed to the surface and away from the cell wall. As previously highlighted for *Sa*SrtB and *Ba*SrtB (Zhang *et al.*, 2004 ▸), the active site of the remaining classes of sortases could be partially buried when anchored onto the bacterial cell wall owing to the active site being positioned on the same plane of the protein as their N-termini.

Analyses of the two class D enzymes further revealed the presence of a unique α-helix structure within the *Cp*SrtD loop that connects the β2 and β3 strands (Fig. 4 ▸ *b*). In contrast, the β2–β3 loop in *B. anthracis* sortase D is uninterrupted, and NOESY spectra suggested that the residues within this loop, as well as the residues in the loop that connects β4 and α1, exhibited resonance line broadening associated with protein oligomerization (Robson *et al.*, 2012 ▸). By comparison, our crystallization studies indicated that *Cp*SrtD is likely to exist in a monomeric form. This notion is supported by gel-filtration analyses, revealing a single peak with a molecular weight that corresponds to a *C. perfringens* sortase D monomer (Supplementary Fig. S1*a*). Additionally, dynamic light-scattering (DLS) experiments to measure the hydrodynamic radius of the enzyme also demonstrated that at the concentration used in the crystallization studies (20 mg ml^−1^) recombinant *Cp*SrtD is likely to be monomeric (Supplementary Fig. S4). It is not known whether the presence of the α-helical structure in *Cp*SrtD plays a role in forcing the enzyme to adopt a monomeric form; further mutational and structural studies would be required to elucidate this. The overall α-helical structures within the *Cp*SrtD crystal structure are indeed distinctive compared with *B. anthracis* sortase D. It is possible that the crystallization buffer conditions used in our studies have provided a more physiological environment (sodium chloride and ammonium acetate salts at about 200 m*M*, compared with the NMR conditions, which were just 20 m*M* HEPES buffer) that allows proper folding of surface secondary structures; further solution studies of the *B. anthracis* sortase D structure under more physiological buffer conditions would be important to provide a better comparative analysis.

Both class D enzymes can also be further differentiated at the catalytic level, where the sortases demonstrated a differential preference towards specific sorting signal motifs. As previously highlighted, the *C. perfringens* sortase D is catalytically active towards the LPQTGS signal motif, but performed inefficiently towards the LPNTGS signal motif (Fig. 3 ▸ *a*), which is highly similar to the LPNTAS motif preferred by the *B. anthracis* sortase D (Robson *et al.*, 2012 ▸). Additionally, both enzymes are catalytically active at different temperatures, with the *C. perfringens* sortase D demonstrating efficient catalysis at 316 K *in vitro* (Fig. 3 ▸ *b*). Interestingly, while the *B. anthracis* sortase D is catalytically active at room temperature (Robson *et al.*, 2012 ▸), our recombinant *C. perfringens* sortase D was highly inefficient when incubated at this temperature *in vitro* (Fig. 3 ▸ *b*). How the LQPTGS signal motif is positioned within the *Cp*SrtD catalytic cleft is unclear; however, comparative analysis with previous structural studies of the *Sa*SrtA–LPAT complex have provided some insights (Zong *et al.*, 2004 ▸). While the environment of the catalytic cleft in *Cp*SrtD is equally rich in hydrophobic residues for substrate binding, it is relatively narrow owing to the position of a small helix within the β6–β7 loop when compared with *Sa*SrtA. As such, it is possible that *Cp*SrtD requires a substrate-induced conformational change to promote proper binding of the LPQTGS motif, but additional structural studies will need to be performed to further elucidate this.

Overall, the *C. perfringens* sortase D reported in this study is structurally and catalytically distinct from the previously reported class D enzyme isolated from *B. anthracis*, suggesting that *Cp*SrtD may represent a new subclass of the sortase D family. Our structural and biochemical analyses suggest further characterization of the biological roles of *Cp*SrtD in promoting spore formation, which will provide further insights into the pathogenesis of foodborne illnesses derived from *C. perfringens* infections. Ultimately, these studies may lead to the development of new antimicrobial agents for controlling foodborne outbreaks associated with this highly pathogenic bacterium.

## Supplementary Material

PDB reference: *C. perfringens* sortase D, 4d70

Supplementary Figures S1-S4.. DOI: 10.1107/S1399004715009219/rr5103sup1.pdf

## Figures and Tables

**Table 1:** Data-collection and refinement statistics for *Cp*SrtD Values in parentheses are for the outer shell.

Data collection	
Space group	*P*2_1_
Unit-cell parameters (, )	*a* = 38.8, *b* = 65.6, *c* = 68.1, = 90.0, = 93.86, = 90.0
Resolution ()	47.21.99 (2.091.99)
*R*_merge_ †	0.074 (0.626)
*R*_p.i.m._ ‡	0.029 (0.252)
CC_1/2_	0.999 (0.843)
*I*/(*I*)	18.0 (3.2)
Completeness (%)	99.5 (97.1)
Multiplicity	7.5 (7.0)
Refinement	
Resolution ()	47.21.99
Unique reflections	22304
*R*_work_/*R*_free_ § (%)	17.6/21.3
No. of atoms	
Total	2621
Protein	2526
Other/ions	0
Water	95
*B* factors (^2^)	
Protein	38.4
Water	37.2
R.m.s. deviations¶	
Bond lengths ()	0.016
Bond angles ()	1.687
PDB code	4d70

† *R*_merge_ = 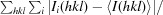 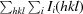.

‡ *R*_p.i.m._ 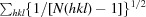 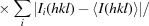 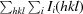.

§ *R*_work_ = 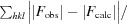 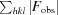 and is calculated using all data; *R*_free_ is the *R* factor based on 5% of the data that were excluded from refinement.

¶ R.m.s.d. is the root-mean-square deviation from ideal values (Engh Huber, 1991 ▸).

## References

[bb1] Brynestad, S. & Granum, P. E. (2002). *Int. J. Food Microbiol.* **74**, 195–202. 10.1016/s0168-1605(01)00680-8 11981970

[bb2] Clancy, K. W., Melvin, J. A. & McCafferty, D. G. (2010). *Biopolymers*, **94**, 385–396. 10.1002/bip.21472 PMC4648256 20593474

[bb3] Collie, R. E., Kokai-Kun, J. F. & McClane, B. A. (1998). *Anaerobe*, **4**, 69–79. 10.1006/anae.1998.0152 16887625

[bb4] Cowtan, K. (2006). *Acta Cryst.* D**62**, 1002–1011. 10.1107/S0907444906022116 16929101

[bb5] Cozzi, R., Prigozhin, D., Rosini, R., Abate, F., Bottomley, M. J., Grandi, G., Telford, J. L., Rinaudo, C. D., Maione, D. & Alber, T. (2012). *PLoS One*, **7**, e49048. 10.1371/journal.pone.0049048 PMC3493515 23145064

[bb6] Cozzi, R., Zerbini, F., Assfalg, M., D’Onofrio, M., Biagini, M., Martinelli, M., Nuccitelli, A., Norais, N., Telford, J. L., Maione, D. & Rinaudo, C. D. (2013). *FASEB J.* **27**, 3144–3154. 10.1096/fj.13-227793 23631841

[bb7] Dramsi, S., Trieu-Cuot, P. & Bierne, H. (2005). *Res. Microbiol.* **156**, 289–297. 10.1016/j.resmic.2004.10.011 15808931

[bb8] Duong, A., Capstick, D. S., Di Berardo, C., Findlay, K. C., Hesketh, A., Hong, H.-J. & Elliot, M. A. (2012). *Mol. Microbiol.* **83**, 992–1005. 10.1111/j.1365-2958.2012.07983.x 22296345

[bb9] Emsley, P., Lohkamp, B., Scott, W. G. & Cowtan, K. (2010). *Acta Cryst.* D**66**, 486–501. 10.1107/S0907444910007493 PMC2852313 20383002

[bb10] Engh, R. A. & Huber, R. (1991). *Acta Cryst.* A**47**, 392–400.

[bb11] Evans, P. R. (2011). *Acta Cryst.* D**67**, 282–292. 10.1107/S090744491003982X PMC3069743 21460446

[bb12] Frankel, B. A., Tong, Y., Bentley, M. L., Fitzgerald, M. C. & McCafferty, D. G. (2007). *Biochemistry*, **46**, 7269–7278. 10.1021/bi700448e 17518446

[bb13] Garcia-Alvarado, J. S., Labbé, R. G. & Rodriguez, M. A. (1992). *Appl. Environ. Microbiol.* **58**, 1411–1414. 10.1128/aem.58.4.1411-1414.1992 PMC195613 1599261

[bb14] Goujon, M., McWilliam, H., Li, W., Valentin, F., Squizzato, S., Paern, J. & Lopez, R. (2010). *Nucleic Acids Res.* **38**, W695–W699. 10.1093/nar/gkq313 PMC2896090 20439314

[bb15] Grass, J. E., Gould, L. H. & Mahon, B. E. (2013). *Foodborne Pathog. Dis.* **10**, 131–136. 10.1089/fpd.2012.1316 PMC4595929 23379281

[bb16] Hendrickx, A. P., Budzik, J. M., Oh, S.-Y. & Schneewind, O. (2011). *Nature Rev. Microbiol.* **9**, 166–176. 10.1038/nrmicro2520 21326273

[bb17] Jacobitz, A. W., Wereszczynski, J., Yi, S. W., Amer, B. R., Huang, G. L., Nguyen, A. V., Sawaya, M. R., Jung, M. E., McCammon, J. A. & Clubb, R. T. (2014). *J. Biol. Chem.* **289**, 8891–8902. 10.1074/jbc.M113.509273 PMC3979406 24519933

[bb18] Jenuja, V. K., Novak, J. S. & Labbe, R. J. (2010). *Pathogens and Toxins in Foods: Challenges and Interventions*, edited by V. K. Jenuja & J. N. Sofos, pp. 53–70. Washington: ASM Press.

[bb19] Kabsch, W. (2010). *Acta Cryst.* D**66**, 125–132. 10.1107/S0907444909047337 PMC2815665 20124692

[bb20] Khare, B., Fu, Z.-Q., Huang, I.-H., Ton-That, H. & Narayana, S. V. L. (2011). *J. Mol. Biol.* **414**, 563–577. 10.1016/j.jmb.2011.10.017 PMC3230703 22033482

[bb21] Kruger, R. G., Otvos, B., Frankel, B. A., Bentley, M., Dostal, P. & McCafferty, D. G. (2004). *Biochemistry*, **43**, 1541–1551. 10.1021/bi035920j 14769030

[bb22] Manzano, C., Contreras-Martel, C., El Mortaji, L., Izoré, T., Fenel, D., Vernet, T., Schoehn, G., Di Guilmi, A. M. & Dessen, A. (2008). *Structure*, **16**, 1838–1848. 10.1016/j.str.2008.10.007 19081060

[bb23] Manzano, C., Izoré, T., Job, V., Di Guilmi, A. M. & Dessen, A. (2009). *Biochemistry*, **48**, 10549–10557. 10.1021/bi901261y 19810750

[bb24] Maresso, A. W., Chapa, T. J. & Schneewind, O. (2006). *J. Bacteriol.* **188**, 8145–8152. 10.1128/JB.01011-06 PMC1698196 17012401

[bb25] Maresso, A. W. & Schneewind, O. (2006). *Biometals*, **19**, 193–203. 10.1007/s10534-005-4863-7 16718604

[bb26] Marraffini, L. A. & Schneewind, O. (2006). *Mol. Microbiol.* **62**, 1402–1417. 10.1111/j.1365-2958.2006.05469.x 17074072

[bb27] Marraffini, L. A. & Schneewind, O. (2007). *J. Bacteriol.* **189**, 6425–6436. 10.1128/JB.00702-07 PMC1951891 17586639

[bb28] Mazmanian, S. K., Liu, G., Ton-That, H. & Schneewind, O. (1999). *Science*, **285**, 760–763. 10.1126/science.285.5428.760 10427003

[bb29] Mazmanian, S. K., Skaar, E. P., Gaspar, A. H., Humayun, M., Gornicki, P., Jelenska, J., Joachmiak, A., Missiakas, D. M. & Schneewind, O. (2003). *Science*, **299**, 906–909. 10.1126/science.1081147 12574635

[bb30] Mazmanian, S. K., Ton-That, H., Su, K. & Schneewind, O. (2002). *Proc. Natl Acad. Sci. USA*, **99**, 2293–2298. 10.1073/pnas.032523999 PMC122358 11830639

[bb31] McClane, B. A. (2007). *Food Microbiology: Fundamentals and Frontiers*, edited by M. P. Doyle & L. R. Beuchat, pp. 423–444. Washington: ASM Press.

[bb32] McCoy, A. J., Grosse-Kunstleve, R. W., Adams, P. D., Winn, M. D., Storoni, L. C. & Read, R. J. (2007). *J. Appl. Cryst.* **40**, 658–674. 10.1107/S0021889807021206 PMC2483472 19461840

[bb33] Murshudov, G. N., Skubák, P., Lebedev, A. A., Pannu, N. S., Steiner, R. A., Nicholls, R. A., Winn, M. D., Long, F. & Vagin, A. A. (2011). *Acta Cryst.* D**67**, 355–367. 10.1107/S0907444911001314 PMC3069751 21460454

[bb34] Naik, M. T., Suree, N., Ilangovan, U., Liew, C. K., Thieu, W., Campbell, D. O., Clemens, J. J., Jung, M. E. & Clubb, R. T. (2006). *J. Biol. Chem.* **281**, 1817–1826. 10.1074/jbc.M506123200 16269411

[bb35] Neiers, F., Madhurantakam, C., Fälker, S., Manzano, C., Dessen, A., Normark, S., Henriques-Normark, B. & Achour, A. (2009). *J. Mol. Biol.* **393**, 704–716. 10.1016/j.jmb.2009.08.058 19729023

[bb36] Newman, J., Egan, D., Walter, T. S., Meged, R., Berry, I., Ben Jelloul, M., Sussman, J. L., Stuart, D. I. & Perrakis, A. (2005). *Acta Cryst.* D**61**, 1426–1431. 10.1107/S0907444905024984 16204897

[bb37] Orsburn, B., Melville, S. B. & Popham, D. L. (2008). *Appl. Environ. Microbiol.* **74**, 3328–3335. 10.1128/AEM.02629-07 PMC2423036 18378644

[bb38] Persson, K. (2011). *Acta Cryst.* D**67**, 212–217. 10.1107/S0907444911004215 21358052

[bb39] Robson, S. A., Jacobitz, A. W., Phillips, M. L. & Clubb, R. T. (2012). *Biochemistry*, **51**, 7953–7963. 10.1021/bi300867t PMC3971844 22974341

[bb40] Scallan, E., Hoekstra, R. M., Angulo, F. J., Tauxe, R. V., Widdowson, M. A., Roy, S. L., Jones, J. L. & Griffin, P. M. (2011). *Emerg. Infect. Dis.* **17**, 7–15. 10.3201/eid1701.P11101 PMC3375761 21192848

[bb42] Seabrook, S. A. & Newman, J. (2013). *ACS Comb. Sci.* **15**, 387–392. 10.1021/co400013v 23710551

[bb43] Shimizu, T., Ohtani, K., Hirakawa, H., Ohshima, K., Yamashita, A., Shiba, T., Ogasawara, N., Hattori, M., Kuhara, S. & Hayashi, H. (2002). *Proc. Natl Acad. Sci. USA*, **99**, 996–1001. 10.1073/pnas.022493799 PMC117419 11792842

[bb44] Sievers, F., Wilm, A., Dineen, D., Gibson, T. J., Karplus, K., Li, W., Lopez, R., McWilliam, H., Remmert, M., Söding, J., Thompson, J. D. & Higgins, D. G. (2011). *Mol. Syst. Biol.* **7**, 539. 10.1038/msb.2011.75 PMC3261699 21988835

[bb45] Spirig, T., Weiner, E. M. & Clubb, R. T. (2011). *Mol. Microbiol.* **82**, 1044–1059. 10.1111/j.1365-2958.2011.07887.x PMC3590066 22026821

[bb46] Stein, N. (2008). *J. Appl. Cryst.* **41**, 641–643.

[bb47] Strong, D. H., Weiss, K. F. & Higgins, L. W. (1966). *J. Am. Diet. Assoc.* **49**, 191–195. 4288386

[bb48] Suree, N., Jung, M. E. & Clubb, R. T. (2007). *Mini Rev. Med. Chem.* **7**, 991–1000. 10.2174/138955707782110097 17979801

[bb49] Suree, N., Liew, C. K., Villareal, V. A., Thieu, W., Fadeev, E. A., Clemens, J. J., Jung, M. E. & Clubb, R. T. (2009). *J. Biol. Chem.* **284**, 24465–24477. 10.1074/jbc.M109.022624 PMC2782039 19592495

[bb50] Ton-That, H., Liu, G., Mazmanian, S. K., Faull, K. F. & Schneewind, O. (1999). *Proc. Natl Acad. Sci. USA*, **96**, 12424–12429. 10.1073/pnas.96.22.12424 PMC22937 10535938

[bb51] Traci, P. A. & Duncan, C. L. (1974). *Appl. Microbiol.* **28**, 815–821. 10.1128/am.28.5.815-821.1974 PMC186831 4374121

[bb52] Weiner, E. M., Robson, S., Marohn, M. & Clubb, R. T. (2010). *J. Biol. Chem.* **285**, 23433–23443. 10.1074/jbc.M110.135434 PMC2906334 20489200

[bb53] Wu, C., Mishra, A., Reardon, M. E., Huang, I.-H., Counts, S. C., Das, A. & Ton-That, H. (2012). *J. Bacteriol.* **194**, 2531–2539. 10.1128/JB.00093-12 PMC3347213 22447896

[bb54] Zhang, R., Wu, R., Joachimiak, G., Mazmanian, S. K., Missiakas, D. M., Gornicki, P., Schneewind, O. & Joachimiak, A. (2004). *Structure*, **12**, 1147–1156. 10.1016/j.str.2004.06.001 PMC2792001 15242591

[bb55] Zong, Y., Bice, T. W., Ton-That, H., Schneewind, O. & Narayana, S. V. L. (2004). *J. Biol. Chem.* **279**, 31383–31389. 10.1074/jbc.M401374200 15117963

