# Supplementary material for: Structural and biochemical analyses of a Clostridium perfringens sortase D transpeptidase
Source: Acta Crystallogr D Biol Crystallogr. 2015 Jun 30;71(Pt 7):1505–13. doi: 10.1107/S1399004715009219 (PMC4498605; doi:10.1107/S1399004715009219)
Supplement: Supplementary file 1 [file d-71-01505-sup1.pdf]

Acta Cryst D
BIOLOGICAL
CRYSTALLOGRAPHY

Volume 71 (2015)

Supporting information for article:

Structural and biochemical analyses of a *Clostridium perfringens* Sortase D transpeptidase

Randy Suryadinata, Shane A. Seabrook, Timothy E. Adams, Stewart D. Nuttall and Thomas S. Peat

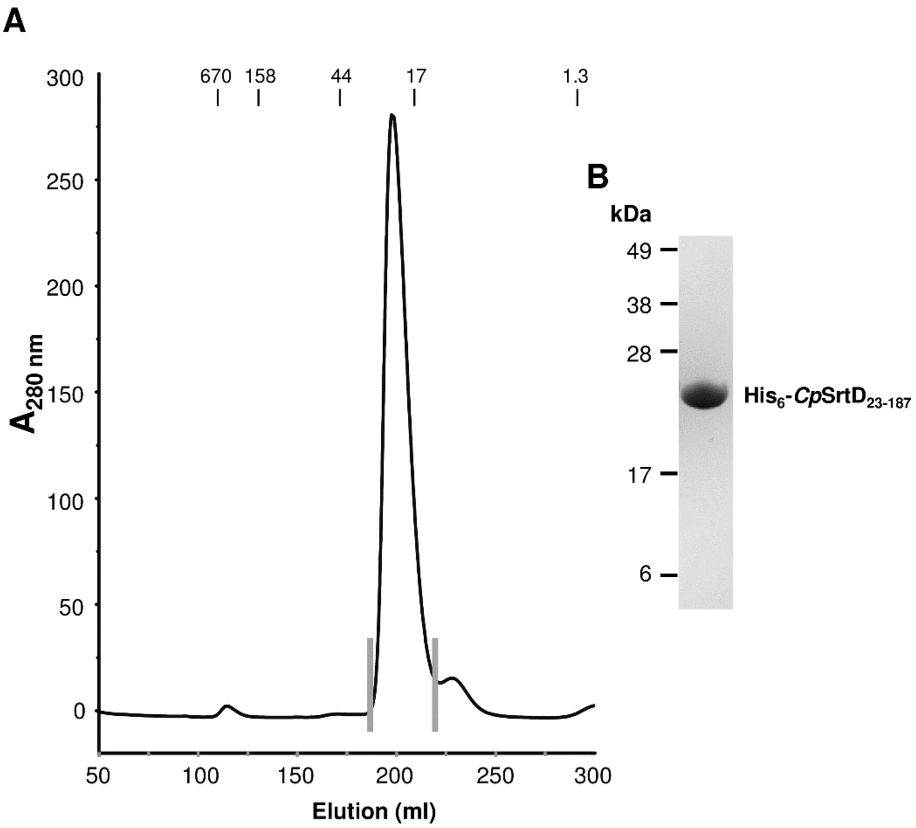

Figure S1 Expression and purification of *Clostridium perfringens* Sortase D. **(A)** Purification of recombinant His_6-*Cp*SrtD$_{23\text{-}187}$ on Superdex 75 26/60 size-exclusion chromatography column. Vertical gray lines indicate the collected monomeric protein fractions. **(B)** SDS-PAGE analysis of purified His_6-*Cp*SrtD$_{23\text{-}187}$ from (A). The approximate molecular weights (kDa) are indicated.

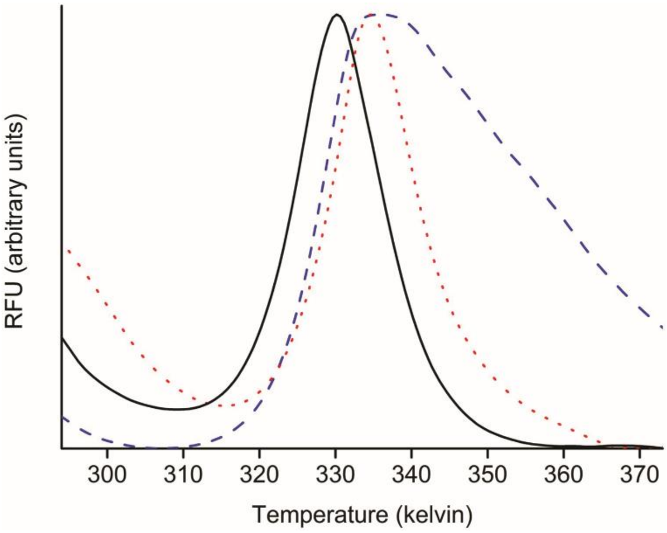

Figure S2 Differential Scanning Fluorimetry (DSF) analysis of recombinant *Cp*SrtD. The melt curve for recombinant *Cp*SrtD in the original buffer (50 mM HEPES pH 7.5, 150 mM NaCl, 5% (*v/v*) glycerol) is represented black solid line. The unfolding profile of the enzyme in 50 mM MES pH 6.5, 200 mM NaCl is shown in blue dashed line, and for comparison, the melt curve for *Cp*SrtD in 50 mM ADA pH 6.5, 200 mM NaCl is shown in red dotted line. The Tm of the original buffer unfolding is at ≈ 325 K while the MES buffer and ADA buffer formulations unfolding are at ≈ 329 K and 331 K, respectively. Recombinant *Cp*SrtD was found to be most stable in a buffer containing 50 mM MES pH 6.5 and 200 mM NaCl. Melt curves are represented as an average of triplicate repeats.

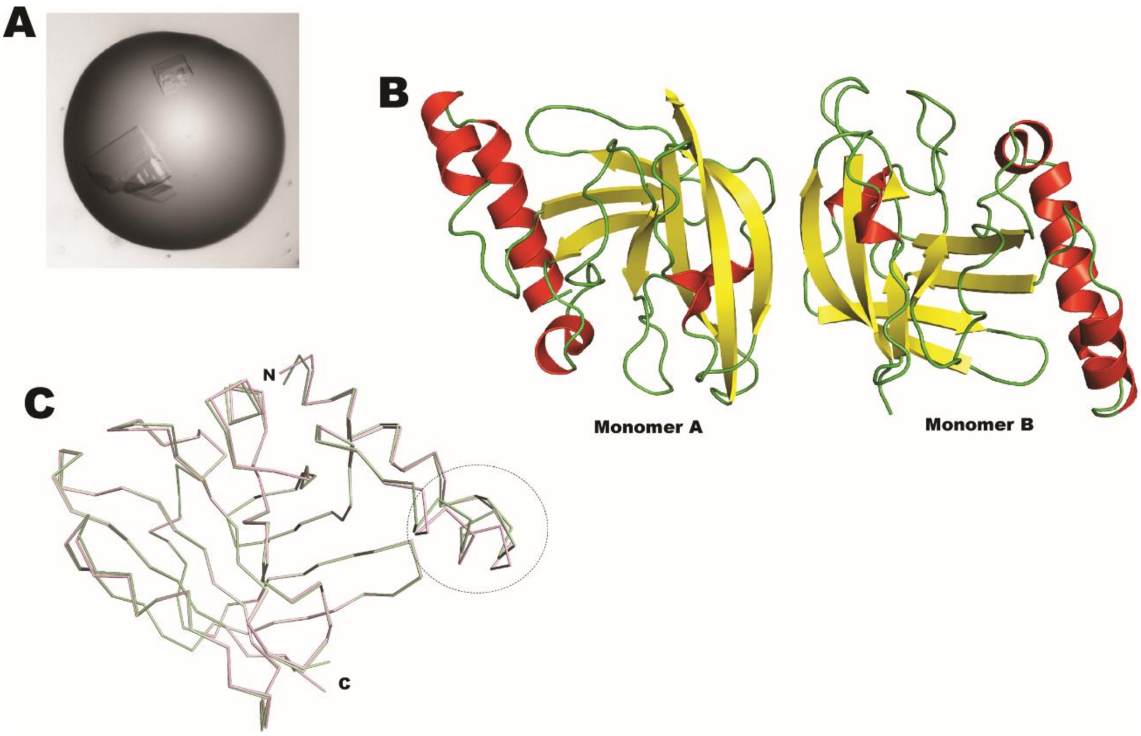

Figure S3 Crystal structure of *C. perfringens* Sortase D. **(A)** Crystals of *C. perfringens* class D Sortase. **(B)** The structure of *Cp*SrtD represented by red 3_{10}- and α-helices, and yellow β-strands, which was determined in the P2_1 space group with two monomers (A & B) in the crystallographic asymmetric unit. **(C)** Superposition of monomers A (green) and B (pink) revealing a minimal conformational difference in the turn within the α1/α2 helix-turn-helix structure (circled). Figures are generated using the program PyMOL (Schrödinger, 2010).

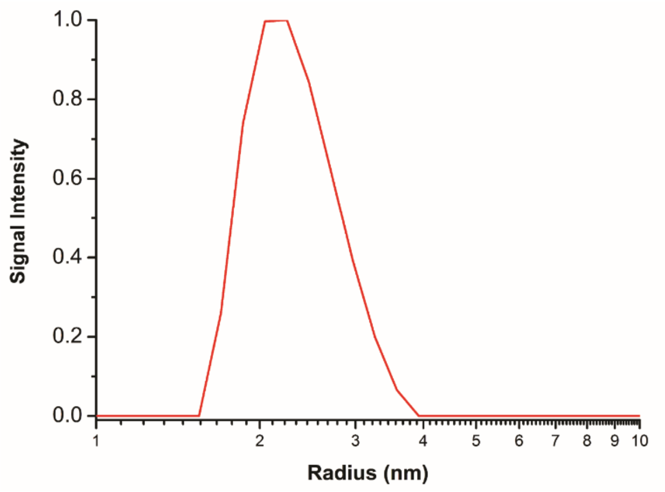

Figure S4 Recombinant *C. perfringens* Sortase D is represented as a monomer *in vitro*. The hydrodynamic size and estimated molecular weights (MW) for a 20 mg ml^{-1} sample of His$_6$-*Cp*SrtD$_{23-187}$ in a buffer containing 50 mM MES pH 6.5 and 200 mM NaCl was measured using batch mode dynamic light scattering in a black 384-well microplate with an optically clear base. The sample returned a radius of ≈ 2.3 nm, corresponding to a MW (kDa) ≈ 24 which supports a monomeric species in solution.
